# Influence of a Water-Based Exercise Program in the Rate of Spontaneous Birth: A Randomized Clinical Trial

**DOI:** 10.3390/ijerph17030795

**Published:** 2020-01-28

**Authors:** Raquel Rodríguez-Blanque, María José Aguilar-Cordero, Ana Eugenia Marín-Jiménez, Ana María Núñez-Negrillo, Antonio Manuel Sánchez-López, Juan Carlos Sánchez-García

**Affiliations:** 1 Research Group CTS1068, Andalusia Research Plan, Junta de Andalucía, San Cecilio Clinical Hospital, School of Nursing, Faculty of Health Sciences, University of Granada, 18071 Granada, Spain; 2 Research Group CTS 367, Andalusia Research Plan, Junta de Andalucía, School of Nursing, School of Health Sciences, University of Granada, San Cecilio Clinical Hospital, 18071 Granada, Sapin; mariajaguilar@telefonica.net; 3 Research Group CTS1068, Andalusia Research Plan, Junta de Andalucía, Quantitative methods for the economics and enterprise, Faculty of Economics and Business Sciences, University of Granada, 18071 Granada, Spain; anamarin@ugr.es; 4 Research Group CTS367, Andalusia Research Plan, Junta de Andalucía, Faculty of Health Sciences, University of Granada, 18071 Granada, Spain; amnunez@ugr.es; 5 Research Group CTS367, Department of Human Motricity and Sports Performance, Faculty of Education Science, University of Sevilla, 41013 Sevilla, Spain; antoniomsanchezlopez@gmail.com; 6 Research Group CTS1068, Andalusia Research Plan, Junta de Andalucía., School of Nursing, Faculty of Health Sciences, University of Granada, 18071 Granada, Spain; jcsg750@gmail.com

**Keywords:** pregnant women, pregnancy, exercise, body mass index, childbirth, partograph

## Abstract

*Introduction*: Many women change their lifestyles and habits when they become pregnant, to avoid potentially harmful effects to themselves and their babies. This is especially so with respect to physical exercise, which is often greatly reduced due to ignorance of the benefits it provides during pregnancy. *Aim*: To evaluate the prevalence of spontaneous birth among women who participated in a water-based physical exercise program. *Methods*: A randomized clinical trial was conducted with a sample of 129 pregnant women (Control Group, CG = 64; Exercise Group, EG = 65). A physical exercise protocol, specifically designed for pregnant women, was created and applied. Those in the EG performed 60 min exercise sessions, three times per week for 17 weeks. The participants in the CG received routine check-ups and advice throughout their pregnancy. *Findings*: The women in the EG presented better results for the onset of spontaneous birth (OR = 2.060 (0.980–4.332)) and for neonate Apgar score of 10 at five minutes (OR = 8.53 (3.60–20.17)). Those who had normal weight at the start of pregnancy achieved better results for spontaneous delivery (OR = 2.099 (1.017–4.335)) than those with overweight/obesity. The rate of caesarean delivery was higher in the women with overweight/obesity (OR = 3.570 ((1.226–10.397)) than in those with normal weight. *Conclusions*: In our study, the women who followed the water-based exercise program gained less weight during pregnancy, which facilitated a better rate of spontaneous, non-instrumental childbirth, together with a better Apgar test score at five minutes.

## 1. Introduction and Background

The WHO recommends maintaining a healthy lifestyle at all stages of life. The new guidelines on prenatal care include 49 recommendations on prenatal care for a positive pregnancy experience [1], including counselling on physical activity.

In 2013, the American College of Obstetricians and Gynecologists (ACOG) reported evidence of an association between excessive weight gain during pregnancy and weight retention after childbirth, together with increased weight of the baby at birth. In addition, an insufficient increase in maternal weight was associated with giving birth to children with a lower body weight [2].

At the ACOG congress in November 2015 [3], it was stated that regular physical exercise has beneficial effects at all times of life, including pregnancy, and the performance of moderate physical exercise, for five days a week, at least 30 min a day, was recommended.

This exercise produces the following beneficial effects during pregnancy: a feeling of well-being, improved sleep and better control of weight gain [4]. The latter question is of fundamental importance, because women with overweight or obesity are at increased risk of experiencing adverse intrapartum and/or neonatal outcomes [5].

Furthermore, physical exercise during pregnancy, in accordance with the ACOG recommendations, facilitates childbirth with fewer obstetric interventions, and reduces the rate of caesarean or instrumental deliveries [6].

Nevertheless, physical exercise during pregnancy has in fact decreased [7], a phenomenon attributed in part to doubts that may arise during pregnancy, in the mother and among healthcare professionals, about whether physical exercise should be performed during pregnancy, and if so, as to the most appropriate type of exercise, and its frequency, intensity and duration [8,9,10].

In this respect, Davies et al. (2003) [11] proposed strength-training programmes of the main muscle groups, to be performed 3–4 times a week, to reinforce the muscles involved in childbirth. Similarly, Bovbjerg and Siega-Riz (2009) [12] argued that having a strong, well-trained abdominal musculature improves the effectiveness of pushing and hence shortens the second stage of labor. With respect to the type of exercise considered most suitable during pregnancy, Artal and O’Toole (2003) [13] and Granath, Hellgren, and Gunnarsson (2006) [14] both concluded that water-based aerobic exercise, compared to that performed in a non-aquatic environment, was less likely to impose an excessive load on the body.

In view of these considerations, we hypothesize that the practice of moderate physical exercise in water, following the SWEP method, between weeks 20 and 37 of pregnancy, is associated with better intrapartum and neonatal outcomes.

Therefore, we aimed to evaluate the prevalence of spontaneous birth among women who participated in a water-based physical exercise program.

## 2. Methods

### 2.1. Ethical Approval

In this open randomized controlled trial, the subjects and researchers were all aware of the intervention. The trial was held in accordance with the 2010 CONSORT standards and it was approved by the Research Ethics Committee of the province of Granada (CEIM Granada), file number 2606.20.13. The study was conducted in compliance with the Declaration of Helsinki, as amended at the 64th WMA General Assembly, Fortaleza, Brazil, in October 2013. Informed consent was obtained from all participants.

The study is registered with the US National Institutes of Health (ClinicalTrials.gov), under the title “Physical Activity in Pregnancy and Postpartum Period, Effects on Women”. Registration number NCT02761967.

### 2.2. Population

The following inclusion criteria were applied: healthy pregnant women, presenting none of the absolute contraindications described in Section 1, Committee Opinion No. 650. American College of Obstetricians and Gynecologists (2015) [3]: heart disease, restrictive lung disease, cerclage for cervical incompetence, multiple gestation with risk of preterm birth, second or third trimester persistent bleeding, preterm labor during current pregnancy, rupture of membranes, preeclampsia, pregnancy-induced hypertension and severe anemia. If a relative contraindication was present, a favorable report was required from the subject’s obstetrician to participate in the study.

If there were signs suggesting that exercise during pregnancy should be suspended, the subject’s gynecologist was consulted about the advisability of continuing with the exercise program.

Women who were less than 12 or more than 20 weeks pregnant, who had taken regular physical exercise during the last 12 months or who had had a multiple pregnancy were excluded from the study. Women who failed to attend at least 80% of the 54 sessions were excluded from the study analysis. The partograph data were compiled in public hospitals in Granada, and therefore any deliveries that took place in hospitals not belonging to the Andalusian Health Service were also excluded from consideration.

### 2.3. Data Sources

The participants’ data were compiled at the corresponding health centers of the Granada Metropolitan Health District, which provide maternity care for the city population and act as reference centers for the province.

The recruitment of participants began in the first half of April 2016, during the ultrasound examination performed in week 12 of pregnancy, and concluded six weeks later, at the end of May 2016.

The researcher responsible for the recruitment contacted 386 potential participants, who were informed of the study conditions. The women who expressed interest in the project were then sent relevant information by email. Of these women, 224 were excluded; 122 presented one or more of the absolute contraindications for physical exercise during pregnancy described in Section 1 of ACOG Document 650, 82 refused to participate (without specifying any reason), and 20 refused to participate for personal reasons such as fear of physical exercise during pregnancy, family responsibilities or difficulty in attending the exercise sessions. The sample, therefore, was composed of 162 pregnant women and after dropouts 140 participants were finally included in the study cohort. The baseline anthropometric data were obtained during week 12, in the first trimester of pregnancy. In week 14, each subject was randomly assigned to one of the study groups, either the Exercise Group (EG) or the Control Group (CG). Each group contained 70 women. Those assigned to the EG were interviewed by the Principal Investigator, who informed them of the expected benefits from attending the exercise sessions.

### 2.4. Intervention

The intervention was carried out in the following way:

• Weeks 20–37 of pregnancy. SWEP program

Taking into account the conclusions drawn in previous research in this field, we developed an exercise program specifically designed for pregnant women. This method is termed SWEP (Study of Water-based Exercise during Pregnancy) and consists of performing moderate physical exercise in an aquatic environment from weeks 20 to 37 of gestation. The sessions take place three times weekly, with a duration of 60 min each (45 min of activity followed by 15 min of relaxation). The sessions are composed of three phases: (a) warm-up; (b) the main phase, divided into aerobic exercise and strength-endurance exercises, designed specifically for pregnant women; (c) stretching and relaxation. The sessions are given in the mornings, after the appropriate intake of food and liquid.

The warm-up phase of the exercise is first general (out of the water) and then specific, as appropriate, in the water.

The aerobic exercise is designed to be performed in a pool 25 m long, where swimming exercises appropriate to the different phases of pregnancy can be performed. The strength and resistance element of the exercise is carried out in a pool 10 m long and 1.50 m deep, and this, too, is appropriate to the duration of pregnancy. In our study, the environmental conditions within the exercise facilities were in accordance with applicable legislation (Decree 23/99 of 23 February, on Sanitary Regulation for Collective-use Swimming Pools, published in the Official Gazette of the Junta de Andalucía No. 36, on 25 March 1999). These regulations stipulate an average water temperature of 27 °C, ambient temperature of 30 °C and average humidity of 72%.

The women in the EG performed moderate physical exercise in water, following the SWEP method [15]. The women in both groups (EG and CG) received verbal and written dietary advice during pregnancy, including the following recommendations:

- Consume foods that are natural, varied, nutritious and light, in quantities to ensure appropriate weight gain, distributed among five or more light meals a day.
- Use salt in moderation (preferably iodized). The oil used should be extra virgin olive oil, and in dressings rather than fried.
- Eat 3–5 servings of fruit a day, plus one of vegetables and salad.
- Consume proteins 4–5 times a week, alternating legumes, eggs, meat and fish.
- Drink 1.5 to 2 litres of water a day.
- Whole-meal bread, pasta, rice and flour are preferable to processed varieties.
- Avoid or reduce the consumption of fried foods, animal fats, precooked meals, sweets and pastries.

Avoid or reduce the consumption of soft drinks, tea, coffee and processed fruit juices.

The CG received the standard recommendations during pregnancy, including guidelines from the midwife on the positive effects of physical exercise. These participants received the usual visits from healthcare providers (midwives, obstetricians and family doctor) during pregnancy, as did those in the EG.

• Weeks 34–36 of pregnancy

The women in each of the study groups were examined by the researchers during weeks 34–36 of pregnancy, and their anthropometric data were recorded.

### 2.5. Variables Studied

The following variables were studied during the intervention:

a. Sociodemographic and anthropometric variables:

Age, gestational age, height and weight. Blood sugar levels were determined in the second trimester.

Weight (kg) was determined using a calibrated scale, during week 12 of pregnancy, termed Weight_Q1, and in week 34, termed Weight_Q3. Height (m) was also measured, using a calibrated metal height rod.

BMI was calculated using the standard formula (Weight kg / Height m^2^) [16,17] in the first trimester, using the data for Weight_Q1 and height, termed BMI_Q1, and in the third trimester, using Weight_Q3 and height, termed BMI_Q3.

The resulting BMI values were then classified as follows, in accordance with WHO guidelines on nutritional status: Low weight <18.50; Normal weight 18.50–24.99; Overweight 25–29.99; Obesity ≥30.00.

Weight gain was evaluated according to the maximum and minimum weight levels recorded between week 12 (the recorded value closest to the pre-gestational value) and week 34, i.e., by subtracting Weight_Q1 from Weight_Q3.

b. Intrapartum and neonatal outcomes:

The progress of labor for each participant was illustrated on a partograph [18], on which the following intrapartum variables were recorded:

- Gestational age (weeks of gestation at the time of delivery).
- Reason for hospital admission
  - Premature rupture of membranes.
  - Delivery in progress or scheduled admission (3 cm dilation and regular contractions, 3–5 contractions every 10 min).
  - Type of delivery conclusion: spontaneous, instrument delivery by forceps, vacuum or spatulas, or surgical act such as caesarean section.

The following neonatal variables were recorded:

- Weight at birth (g).
- Apgar test to determine the neonate’s tolerance at birth (score at 1 min) and adaptation to extrauterine life (score at 5 min), according to heart rate, respiratory effort, muscle tone, response to stimuli and skin tone. The test score ranges from 0–10. Scores of 7–9 are considered normal, <7 indicates need for resuscitation. The lower the score, the greater the neonatal depression and the greater the need for more advanced resuscitation maneuvers.
- Resuscitation maneuvers performed (drying and type of resuscitation). Apgar score >7, the neonate should only be dried. With scores <7, resuscitation should be initiated.

### 2.6. Sample Size

The sample size was calculated taking into account the findings of previous research in this area, based on a program of physical exercise for pregnant women. The main variable considered was the prevalence of spontaneous birth in the study cohort. According to the results obtained by Price et al. [19], the prevalence of spontaneous birth should increase from 61.2% to 87% with the practice of physical exercise during pregnancy. To obtain a power of 80% to detect differences in the null hypothesis test H_0_: μ_1_ = μ_2_ by applying a chi-square test, for a 5% level of significance, we calculated that 45 women per group, 90 in total, would need to be included in the study.

Study group assignment was random, probabilistic, without replacement and open label. The participants and the researchers were all fully aware of the different phases of the intervention. The identification data of each participant who met the inclusion criteria were noted on a ticket by one of the researchers. Each ticket was placed in an opaque envelope and each envelope in a container. Once all the envelopes were in the container, the Principal Investigator removed 81, which were assigned to the EG, and the remaining 81 were assigned to the CG.

### 2.7. Statistical Analysis

As inferential analysis, Student’s t-test was performed to compare the parametric variables, and the Mann–Whitney U test was applied to the non-parametric variables. The Pearson chi square test was used to compare intrapartum and neonatal outcomes between the groups. For each group, the odds ratios were calculated for spontaneous delivery, for the Apgar score at five minutes, and according to first-trimester BMI, for non-spontaneous and caesarean section delivery, with 95% confidence intervals. All statistical analyses were performed using IBM SPSS 19 (SPSS Inc., Chicago, USA) statistical software. The level of significance assumed was *p* < 0.05.

To compare the intrapartum and neonatal outcomes by BMI, the variable was classified into three categories; BMI ≤ 18.5 = Low weight; BMI 18–25 = Normal weight; BMI ≥ 25 = Overweight/Obesity (for greater statistical power, the latter two categories were combined).

## 3. Results

**Figure 1:**
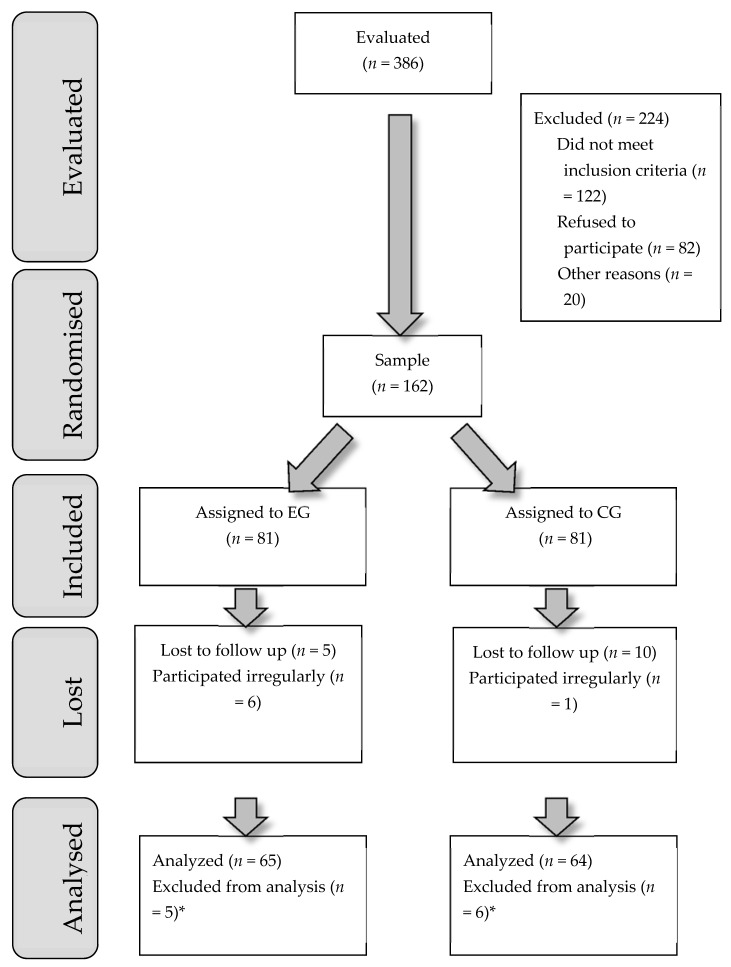
Flow diagram. * The delivery did not take place at the Granada Hospital Complex.

The recruitment process for the study samples is shown in Figure 1.

The study samples analyzed consisted of 64 women in the CG and 65 in the EG. The participants’ ociodemographic and anthropometric characteristics are shown in Table 1.

The average age of the participants, in each of the study groups, was around 34 years, ranging from 22–46 years in the CG and 24–45 years in the EG. Blood glucose levels, determined in the second trimester, were higher in the CG (120.49 ± 1.78 vs. 119.87 ± 2.10), but the difference was not statistically significant (*p* = 0.075). The women’s average weight during the first trimester, in both groups, was almost 68 kg. By the end of the third trimester this had increased to 79 kg in the CG, with an average gain of 11.17 kg, and to 75.35 kg in the EG, with a gain of 8.28 kg.

The results obtained from this analysis show that for the sociodemographic and anthropometric variables age, height, weight_Q1 and BMI_Q1, the pattern of the variances was the same in the CG and in the EG. Similarly, Levene’s test showed all the variances to be equal. There was a significant difference, however, for the weight gain variable, which was greater in the CG than in the EG. Weight_Q3 and BMI_Q3 were also higher in the CG than the EG, although these differences did not reach statistical significance.

The median birth Weight was 3460 g (first quartile = 3207.5 g, third quartile = 3770.0 g) in the CG and 3250 g (first quartile = 2955.0 g, third quartile = 3572.5 g) in the EG, and the difference was statistically significant (*p* = 0.011).

Chi-square tests were used to compare the intrapartum and neonatal outcomes, revealing significant differences between CG and EG for the Apgar score at five minutes, *p* < 0.001. An Apgar score of 10 was presented by 32.5% of the CG and by 67.5% of the EG. Of the neonates with a score of 8–9, 80.4% were from the CG and 19.6% from the EG. We conclude there were significant differences between the study groups for spontaneous birth. Of the women with non-spontaneous birth, 61.4% were from the CG and 38.6% from the EG.

Table 2 shows the odds ratios obtained for spontaneous birth and for the Apgar score at five minutes, by study group.

The results shown in Table 2 highlight the fact that the neonates born to the women in the EG were more likely to have a five-minute Apgar score of 10 than those born to the women in the CG.

According to the BMI classification obtained for the study sample, none of the women were classed as low weight; 62% were normal weight and 38% presented overweight/obesity.

The chi-square test was used to determine whether BMI was associated with the intrapartum and neonatal outcomes. The results obtained showed that both spontaneous delivery and caesarean section were affected by the mother’s BMI in the first trimester (BMI_Q1), *p* = 0.065 and 0.029, respectively.

When the analysis was performed with the BMI variable without categorising the results obtained, using Student’s t-test, similar results were obtained, i.e., both spontaneous birth and caesarean section were associated with the mother’s BMI. For spontaneous birth (*p* = 0.026), the average BMI was 24.078 (SD = 4.02), and in other cases it was 25.844 (SD = 4.89). The women who required a caesarean section had a mean BMI of 29.982 (*p* = 0.000; SD = 5.23) while for those who did not, the mean BMI was 24.185 (SD = 4.007).

The latter results are also reflected in those obtained when the mother’s weight during the first trimester is considered. Thus, the t-test was significant for spontaneous birth (*p* = 0.031) and for caesarean section (*p* = 0.000). The average weight of the women with a spontaneous birth was 65.489 kg (SD = 12.06), versus 70.224 kg (SD = 12.36) among those requiring instrumental delivery or a caesarean section. Among those requiring a caesarean section, the average weight was 77.912 kg (SD = 12.29) versus 65.887 kg (SD = 11.63) for those who did not. Table 3 shows the odds ratios for the results for spontaneous birth versus caesarean section, according to the mother’s weight (normal or overweight–obesity) in the first trimester.

The proportion of women who did not give birth spontaneously was 2.10 times higher among those with overweight-obesity. Similarly, the proportion of women requiring a caesarean section was 3.570 times higher among those with overweight.

With respect to the association between weight gain during pregnancy and intrapartum and neonatal outcomes, the women in the CG (i.e., those who did not perform regular moderate physical exercise in water) recorded a higher weight gain. Table 4 shows the relationship between this weight gain and the intrapartum and neonatal outcomes.

From the results shown in Table 4, we conclude that the women who gained most weight during pregnancy were most likely to require instrumental delivery. An Apgar score of 10 at five minutes was more likely for the babies born to the women in the EG, who gained less weight during pregnancy, while those in the CG were more likely to record an Apgar score of 8 or 9. Although the differences were not statistically significant, clinical differences were observed between the two groups; thus, the babies were more likely to need resuscitation (rather than standard care) when their mothers gained more weight during pregnancy.

There was no association between the age of the mother, the duration of pregnancy or the weight of the baby at birth with the intrapartum or neonatal outcomes.

## 4. Discussion

Our study results show that the women who followed the SWEP method were more likely to have a spontaneous delivery and their babies had a higher Apgar score at 10 min, than those in the CG.

During pregnancy, women should be advised to optimize their lifestyle habits and to avoid excessive weight gain. Healthcare professionals should be given specific guidelines on advising their patients to perform physical activity during pregnancy (according to the American College of Obstetrics and Gynecology), in order to reduce the possibility of complications in childbirth, among other outcomes. Barakat et al. (2009) [20] cite numerous studies corroborating the contribution made by prenatal exercise to preventing excessive weight gain during pregnancy.

Crane et al. (2009) [21] reported that women whose weight gain during pregnancy was in line with medical recommendations had a lower rate of adverse outcomes in childbirth. With respect to the association between weight gain during pregnancy and intrapartum and neonatal outcomes, in our study, the women in the CG (i.e., those who did not perform regular moderate physical exercise in water) recorded a higher weight gain. We conclude that the women who gained most weight during pregnancy were most likely to require instrumental birth (see Table 4). Cavalcante et al. (2009) [22] observed that most of the 34 low-risk sedentary pregnant women in the EG who had performed aerobic exercise in water since week 18-20 of pregnancy had normal vaginal deliveries. When the BMI analysis was performed without categorizing the results obtained, using Student’s t-test, similar results were obtained, i.e., both spontaneous birth and caesarean section were associated with the mother’s BMI. With respect to the need for resuscitation, although the differences were not statistically significant, the newborns were more likely to need resuscitation (rather than standard care) when their mothers gained more weight during pregnancy [23,24].

In another study in this field, Nascimento et al. (2011) [25] analyzed a group of pregnant women with overweight and obesity. Those in the EG performed 40 min sessions of moderate intensity in their homes, and gained less weight during pregnancy than those in the CG, who did not perform any such program of physical exercise. These data, with regard to women with overweight, are similar to our own (see Table 1). However, we studied the differences between two categories of BMI: on the one hand, women with normal weight, and on the other, the combined category of overweight/obesity. In this respect, statistical significance was observed in the BMI categories studied. There was a significant difference for the weight gain variable, which was greater in the CG than in the EG. There were also differences for the variables weight and BMI in the third trimester, which in both cases were greater for the CG.

Torres-Luque et al. (2012) [26] studied 15 pregnant women who took part in a programme of moderate physical exercise in water, consisting of three 50–60 min sessions per week for six weeks. The results obtained in this study revealed no statistically significant differences between the start and end measurements for the study variables. However, in our own study, the sample was much larger and the exercise program had a duration of 17 weeks. Under these circumstances, statistically significant results were obtained between the two groups, and within the EG over time. Thus, these results strongly indicate the advisability of performing physical exercise during pregnancy, as part of a program supervised by specialists, in order to control weight gain during this period.

Barakat et al. (2006) [27], studied two groups of pregnant women: one performed moderate aerobic exercise and the other did not. Although no statistically significant differences were found, these authors reported that the EG women presented a lower average weight gain and moreover, the weight of the neonates at birth was lower than in the CG. This finding is in line with our study results, which reflected statistically significant differences in both variables.

However, contrasting research findings have also been reported. Thus, Perales et al. (2016) [28] studied a group of pregnant women who performed a physical exercise program for three days a week. Although the weight gain in this group during pregnancy was 11.6 kg, compared to 12.6 kg in the CG (p = 0.06), the difference was not statistically significant. This lack of significant impact may be because the exercises focused on resistance, toning, aerobic dance and pelvic floor strengthening. On the other hand, the SWEP method was designed specifically for the pregnant woman and is based on a combination of aerobic and moderate-intensity strength/resistance exercises.

Our literature review also included studies of how BMI affects intrapartum and neonatal outcomes, such as Foo et al. [5], Athukorala et al. [25], Kim et al. [26] and Denison et al. [27]. All of these papers concluded that women with overweight or obesity were more likely to require induced labor or a caesarean section, compared to women with a lower BMI, which corroborates our findings that pregnant women with normal weight are less likely to require the induction of labor or an emergency caesarean section. The previous studies also observed that babies born to women with a higher BMI were more likely to require neonatal resuscitation and/or admission to the neonatal intensive care unit, and to have a lower Apgar score at five minutes. Our study results showed that women who followed the SWEP method had a greater probability of having a baby with an Apgar score of 10 at five minutes, compared to sedentary women, and also were more likely to have a spontaneous birth.

## 5. Conclusions

Participation in a program of physical exercise during pregnancy, based on the SWEP method, enhances the control of weight gain, and thus promotes a higher rate of spontaneous birth and a lower rate of instrumental deliveries and of caesarean sections. Women whose weight gain during pregnancy is in line with recommendations are more likely to have a baby with an Apgar score of 10 at five minutes.

Furthermore, women whose BMI is in the normal-weight range at the start of their pregnancy are more likely to give birth spontaneously than those with overweight or obesity before pregnancy. An appropriate weight gain during pregnancy facilitates a more physiological birth, while women who present a greater weight gain during pregnancy are more likely to require instrumental birth.

## Figures and Tables

**Table 1:** Sociodemographic and anthropometric data.

	CG (*n* = 64)						EG (*n* = 65)				
	Mean	Median	SD	Min.	Max.	*p*	Mean	Median	SD	Min.	Max.
**Age**	33.47	33.00	5.18	22	46	0.136	34.74	35.00	4.41	24.00	45.00
**Height**	1.65	1.65	0.05	1.50	1.77	0.604	1.65	1.64	0.06	1.52	1.78
**Weight_Q1**	67.88	67.00	12.58	48.00	107.00	0.710	67.07	66.00	12.23	47.00	101.00
**Weight_Q3**	79.05	79.00	11.64	55.00	114.00	0.079	75.35	74.00	12.11	53.00	110.00
**Weight_gain**	11.17	10.75	3.47	2.50	21.00	0.000	8.28	8.50	2.83	1.50	14.00
**BMI_Q1**	24.93	24.01	4.80	18.69	39.56	0.774	24.70	23.89	4.16	18.83	38.45
**BMI_Q3**	29.03	28.34	4.45	22.30	43.11	0.092	27.76	27.05	4.03	20.42	39.30

CG, control group; EG, exercise group; Weight_Q1, weight in the first trimester; Weight_Q3, weight in the third trimester; Weight_gain, Weight_Q3 less Weight_Q1; BMI_Q1, BMI in the first trimester; BMI_Q3, BMI in the third trimester.

**Table 2:** Neonatal outcomes by group.

		EG	CG	OR	95% C.I.
Apgar 5 min	10 points	56 (67.5%)	27 (32.5%)	8.53	[3.60, 20.17]
	8–9 points	9 (19.6%)	37 (80.4%)		
Spontaneous birth	Yes	48 (56.5%)	37 (43.5%)	2.06	[0.98, 4.33]
	No	17 (38.6%)	27 (61.4%)		

**Table 3:** Odds ratios according to first-trimester BMI.

		Normal Weight	Overweight–Obesity	OR	95% C.I.
Spontaneous birth	Yes	52 (69.3%)	23 (30.7%)	2.10	[1.017, 4.335]
	No	28 (51.9%)	26 (48.1%)		
Caesarean section	Yes	6 (35.3%)	11 (64.7%)	3.57	[1.226, 10.397]
	No	74 (66.1%)	38 (33.9%)		

**Table 4:** Student’s t-test results for weight gain vs. intrapartum and neonatal outcomes.

		Mean (kg)	Sig.	95% C.I for Mean Difference
Non-spontaneous labor	Yes	10.155	0.300	[−1.94, 0.61]
	No	9.485		
Spontaneous birth	Yes	9.373	0.190	[−0.41, 2.03]
	No	10.186		
Instrumental delivery	Yes	10.899	0.013	[−2.97, −0.35]
	No	9.237		
Caesarean section	Yes	8.635	0.170	[−0.54, 3.02]
	No	9.877		
Resuscitation	Yes	10.358	0.104	[−2.27, 0.22]
	No	9.332		
Drying	Yes	9.332	0.104	[−0.25, 2.27]
	No	10.358		
Apgar score at 5 min.	Yes	10.979	0.002	[0.75, 3.19]
	No	9.012		
